# Hepatobiliary complications from ruptured silicone breast implants – a comprehensive literature review

**DOI:** 10.3205/000292

**Published:** 2021-05-25

**Authors:** Joshua Agilinko, Dharshanan Raj, Ken Vin Wong, Daniele Fanelli, Nicklaus Ng, Bertrand Agilinko, Mohammad Hasan

**Affiliations:** 1 Department of General Surgery, North Middlesex University Hospital, London, United Kingdom; 2 Department of General Surgery, Aberdeen Royal Infirmary, Aberdeen, United Kingdom; 3 University of Aberdeen School of Medicine and Dentistry, Aberdeen, United Kingdom; 4 Sandema District Hospital, Sandema, Ghana

**Keywords:** literature review, silicone implants, hepatobiliary, complications

## Abstract

Cronin and Gerow first introduced silicone breast implants in 1962; they now serve as first-line for breast augmentation. Breast augmentation is effective in restoring both physical and psychological well-being in women post-mastectomy.

Many studies in the literature on complications of silicone breast implant rupture focus on lymphomas and capsular contractures. Only a few studies discuss the hepatobiliary complications.

By reviewing the literature over the past 30 years, the authors aim to analyse the clinical presentation, diagnostic findings, as well as management outcomes amongst women with ruptured silicone implant-related hepatobiliary complications. To the best of our knowledge, this is the first comprehensive review on this topic.

## Introduction

Societal expectations and evolving importance of the ideal female body has led to a rise in the practice of breast augmentation surgeries.

In 1895, Vincenz Czerny was the first surgeon to attempt breast augmentation surgery by transferring lumbar lipoma to the breast tissue [1].

Concerns over first-generational breast implants like polytetrafluoroethylene stemmed from their thick shells, leading to higher rates of capsular contractures and subsequent rupture and leakage of implant material [2].

Cronin and Gerow introduced silicone breast implants in 1962; they are now the first line devices in breast augmentation. Their smooth-textured shells offer a stable spatial filling post-mastectomy [3].

Since then, silicone implants have undergone several modifications to reduce the risk of leak, which causes local and systemic post-surgical/procedure complications.

Proposed mechanisms for silicone implant rupture include trauma to implant and shell swelling; the latter a phenomenon explaining a decrease in shell strength due to migration of silicone fluid from the gel [4].

The focus of many studies on complications of silicone breast implant rupture centre on lymphoma and capsular contracture [5], [6]. Only few studies discuss the hepatobiliary complications [7], [8], [9].

By reviewing the literature over the past 30 years, the aim of this review is to analyse the clinical presentation, diagnostic findings, as well as management and treatment outcomes amongst adults with ruptured silicone implant-related hepatobiliary complications.

## Methods and materials

### Search strategy

**Figure 1:**
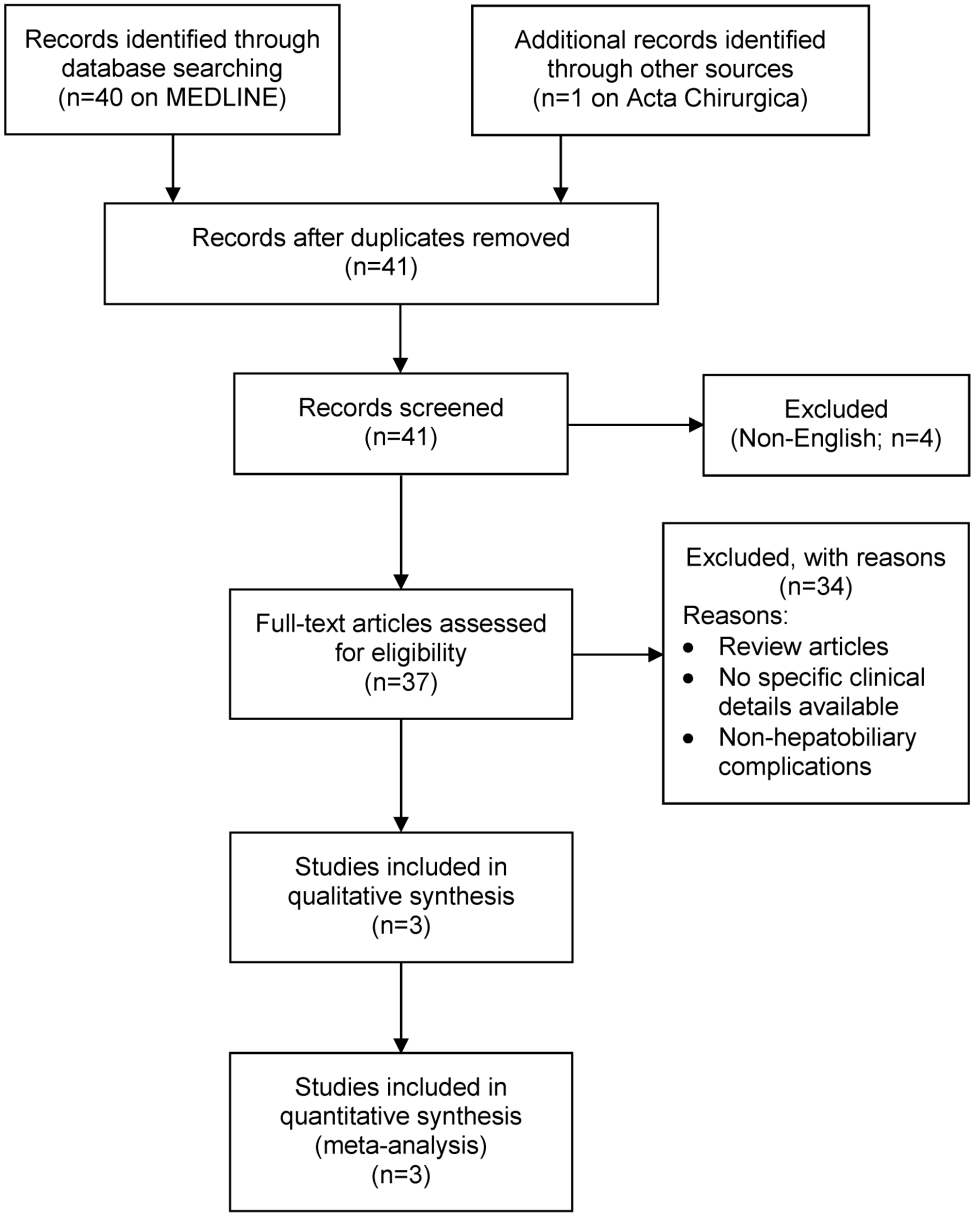
PRISMA flow diagram

This literature review was performed according to the Preferred Reporting Items for Systematic Reviews and Meta-Analyses (PRISMA) guidelines [10] (Figure 1 (Fig. 1)).

The literature search was performed using Pubmed, Medline and Embase in July 2020. The search terms included: (“silicone” AND “breast”) AND (“hepatobiliary” OR “liver” OR “hepatic”). The titles and abstracts were screened by DR and KW while NG independently verified inclusion of the articles. Any discrepancies were resolved by consulting the lead author (JA). Inclusion criteria included:

- silicone implants,
- hepatobiliary complications,
- English language.

Exclusion criteria were:

- saline or other implant type,
- non-hepatobiliary complications (local or systemic).

These criteria were applied throughout the titles and abstract screening stage and the full-text articles reviewing process.

### Data extraction and quality assessment

Quantitative aspects were represented by demographics, type and severity of hepatobiliary complications.

Each study was assigned a level of evidence according to the Oxford (UK) CEBM Levels of Evidence. Quality assessment was conducted with the JBI critical appraisal checklist for case reports [11]. This tool considers the quality of description of demographic characteristics, patients’ history, clinical course and investigations. Only case reports achieving a minimum score of 5 out of 8 were included.

We could not perform a formal meta-analysis considering the absence of randomized controlled trials and cohort studies. Instead, we conducted a critical appraisal of the available literature, describing patients’ characteristics, clinical course, investigations and treatment.

### Statistical analysis

Descriptive statistics were performed with the numbers available. Weighted means and standard deviations (SD) were calculated for data regarding demographics and complications severity categories.

When SD were not directly provided, these were calculated with the equation [max range–min range/4].

## Results

The search strategy revealed paucity of literature in the research topic, with 3 papers (4 case reports) describing hepatobiliary complications after ruptured silicone breast implants [7], [8], [9]. A paper by Tan et al. (titled: hepatobiliary complications following breast implants: a case report and literature review) was excluded, as the paper has since been retracted from literature by the time the authors of this paper finished writing.

The 3 studies (4 case reports) included in our final review are summarised in the Case summary and Table 1 (Tab. 1).

## Case summary

### Age and ethnicity

The mean age in years at the time of presentation was 50 (range:38–58). Information about ethnicity was available in all the papers: 3 Caucasians [8], [9]; and 1 Hispanic [7].

### Type of implant and laterality

A polyurethane cover, filled with synthetic thermostable rubber of low molecular structure was used in 1 patient [9]. The type of implant material was not reported in the other studies. In the 3 cases (2 studies) reporting on laterality, the implants were inserted bilaterally [7], [9] and unilaterally (left breast) in 1 case [7].

### Duration and presenting complaint

The mean interval between implant insertion and onset of symptomatology was 18.2 years (range 10–30; SD 8.05).

Abdominal pain (right upper quadrant and epigastric pain) was the most common presenting complaint [7], [9]. 1 patient presented with symptoms of chronic liver disease; pruritis and lethargy [7] and weight loss [8].

### Medical history

1 patient had Sjogren’s disease with positive anti-Ro antibodies [7]. Another patient had a history of iron-deficiency anaemia secondary to menorrhagia requiring regular iron supplements [8]. Quicke’s oedema was reported in a patient with recurrent episodes of facial swelling [9].

### Biochemistry and haematology

All the patients had deranged LFTs on admission. Alanine transaminase (ALT) and aspartate aminotransferase (AST) were commonly raised [7], [8]. Patients presenting much later after implants were inserted presented with greater LFTs derangement. In 2 patients, mild anaemia was demonstrated on haematology findings [7], [9]. In 1 patient, inflammatory markers such as C-reactive protein and erythrocyte sedimentation rate were raised [8].

### Virology and serology

A test for viral hepatitis was normal in 3 patients [7], [8]. In the same patients, antinuclear (ANCA) and antimitochondrial (AMA) antibodies were normal.

### Histology and immunohistochemistry

Histology and immunohistochemistry findings were diagnostic for silica-induced hepatobiliary pathology in all the studies. The most common liver biopsy finding was granulomatous material within the liver parenchyma; 1 patient had necrotic material [7] and 2 were non-necrotic [8]. The foamy, multi-vacuolated granulomatous material compatible with silicone was reported in the former patient [7]. In 2 cases, trichrome stain and energy dispersible spectroscopy (EDS) were used [8]; with the former reporting a “swiss cheese-like” pattern, consistent with the appearance of a silicone granuloma.

### Imaging

In the patient who underwent liver ultrasound, there was intra-hepatic biliary dilatation and a hypoechogenic focus within the right liver lobe, most likely a silicone deposit [9]. In the patients who had CT scan performed (n=2), a small hepatic cyst [8] and gastrosplenic varices [8] were reported.

The most common MRI finding was cholecystitis. MRI of the breast in 1 patient was diagnostic of ruptured breast implant, as an aetiology of their symptoms [8].

### Treatment and outcome

Treatment and outcomes were only reported in 3 patients [7], [8]. 1 patient underwent cholecystectomy for chronic cholecystitis [7], 1 patient was lost to follow-up [8], and no further management was initiated in 1 patient [8].

## Discussion

Augmentation mammoplasty is among the most frequently performed operations in United Kingdom. The goal is to improve patients’ quality of life based on physical appearance and self-esteem. Satisfaction rates of up to 95% have been reported in studies reviewing quality of marital life following breast augmentation surgeries [12].

Cronin and Gerow first introduced silicone implants in 1962 [3]. Since then, they are first line in breast augmentation surgeries. Their thin shells and inert nature allows them to function as spatial fillers as well as having a low risk of local and systemic reactions. Despite this, concerns over their use, including risk of lymphoma and capsular contractures have been well documented in literature [5], [6]. As our review shows, only few studies discuss the hepatobiliary complications associated with their rupture [7], [8], [9].

Breast implant rupture and leakage can potentially cause foreign body granulomatous reactions and deposition of silica particles in the liver parenchyma [13].

In a study of 149 patients, Collis and Sharpe concluded that implant rupture begins at around 6 years and by 13 years, 11.8% of implants have ruptured. The median life expectancy of silicone implants is reported to be approximately 10–16 years [14]. In our review, the median age from time of insertion of implants to abdominal symptomatology was 18.2 years.

The link between silicone implants rupture and hepatobiliary disease is not well understood. Like other inflammatory and connective tissue diseases, silicone breast implants may act as a foreign body and elicit autoantibody production in the liver parenchyma after leakage. The term “autoimmune syndrome in adjuvants”(ASIA) was coined by the immunologist Shoenfield et al. to suggest such a probable link [15]. A case of sarcoidosis in a patient with silicone breast implant rupture has also been reported in the literature [16]. One patient in our review had Sjögren’s syndrome with positive serum anti-Ro antibodies.

Further to the above, current research suggests that the liver is a common site for silicone particles deposition [7], [8]. They tend to deposit within portal tract cells activating macrophages and Kupffer cells, resulting in chronic hepatitis. Symptomatology of acute on-chronic liver disease, chronically elevated liver enzymes are hallmarks of hepatobiliary complications of ruptured silicone implants. Histopathological evidence of granulomas and silica particles on electron microscopy and energy dispersive spectroscopy has also been reported in previous studies [7], [8], [9].

The activation of macrophages explains the findings of granulomas (epithelioid/activated macrophages). The necrotic nature of some of these granulomas is greater as more Kupffer cells are produced and function to cause further breakdown of cells within the liver parenchyma.

Mechanisms for the actual rupture of breast implants have been extensively studied.

Silicone implant rupture and subsequent leakage is likely a multi-factorial process. Various mechanisms which have been proposed include trauma to the implant and the so-called ‘shell swelling’ phenomenon [4]. Shell swelling occurs after placement of implants, and it is described as a decrease in shell strength due to migration of silicone fluid from the gel into the shell. Brandon et al. postulated that failure at the site of implants fold, as an aetiology of implant rupture [17]. As such, implant folding is more common in the presence of capsular contracture of prolonged duration.

Concerns over first-generational breast implants like polytetrafluoroethylene stemmed from their thick shells, leading to higher rates of capsular contractures and breast distortion [2]. It is likely that the lower contracture rates associated with the thin-shelled silicone implants reduces the rate of leakage. Spear and Murphy reported an overall rupture rate of 13% in fourth generation silicone implants [18].

Intracapsular silicone implant leak is relatively easier to diagnose. Changes in breast shape and size, palpable lumps and pain often give initial diagnostic clues [19]. Contrarily, extracellular implants leak (which often leads to systemic complications) do not manifest so clearly, with clinically significant signs or reported symptoms, often classified as ‘silent’ [20]. This makes diagnosis and subsequent management challenging. It is unsurprising, therefore, that only 50% of the patients in our review presented with abdominal pain, and relying on this to diagnose a hepatobiliary complication of implant rupture is not clinically sufficient. It is perhaps more helpful to consider abdominal pain in the context of symptomatology of chronic liver disease. Symptoms and signs of chronic liver disease including pruritus, weight loss and lethargy were seen in the patients in our review. Additionally, physical examination is an important step in the evaluation of patient symptomatology. However, the aforementioned study conducted by Hölmich and colleagues, reviewing the role of physical examination implant rupture diagnosis, reported a modest sensitivity and specificity of 30% and 88% respectively [20].

MRI is widely regarded as the first-line imaging modality in diagnosing intracapsular implant rupture, with a specificity of more than 90% in evaluating rupture. Classic findings include the linguini and tear drop signs [21]. Such findings were noted in one patient in our review with ruptured left breast implant 10 years after insertion. This has led to The Food and Drug Agency in the US recommending MRI screening of female patients with silicone implants every 2–3 years [22]; this could be adopted globally as a follow-up and prognostic investigative tool. Ultrasound can also be utilised in detecting implant ruptures. One patient in our review showed signs of a hypoechogenic focus within the right liver lobe, suggestive of silica deposit. However, ultrasound has a lower sensitivity and negative predictive value in extracapsular rupture detection [23].

Based on previous studies on liver fibrosis and granulomatous diseases, liver biopsy is the gold standard for diagnosis of liver diseases [24]. Histology and immunohistochemistry of sampled liver cells was diagnostic in all 4 patients included in our review. Granulomas, both necrotic and non-necrotic, were common findings, with the former highlighting the destructive nature of silica particles deposition in the liver parenchyma.

Further to this, the Masson trichrome stain is widely used in liver studies to distinguish collagenous tissue from muscle cells [25]. This was important in the diagnostic work-up in the patients in our review, contributing to the diagnosis of silica particles in 2 of the studies.

Definitive treatment of silicone implant rupture requires removal of implant. Remission of sarcoidosis has been reported in a patient following removal of the silicone gel [16].

Additional treatment involve targeted treatment; such as cholecystectomy, which one patient underwent in our study.

## Notes

### Competing interests

The authors declare that they have no competing interests.

## Figures and Tables

**Table 1:**
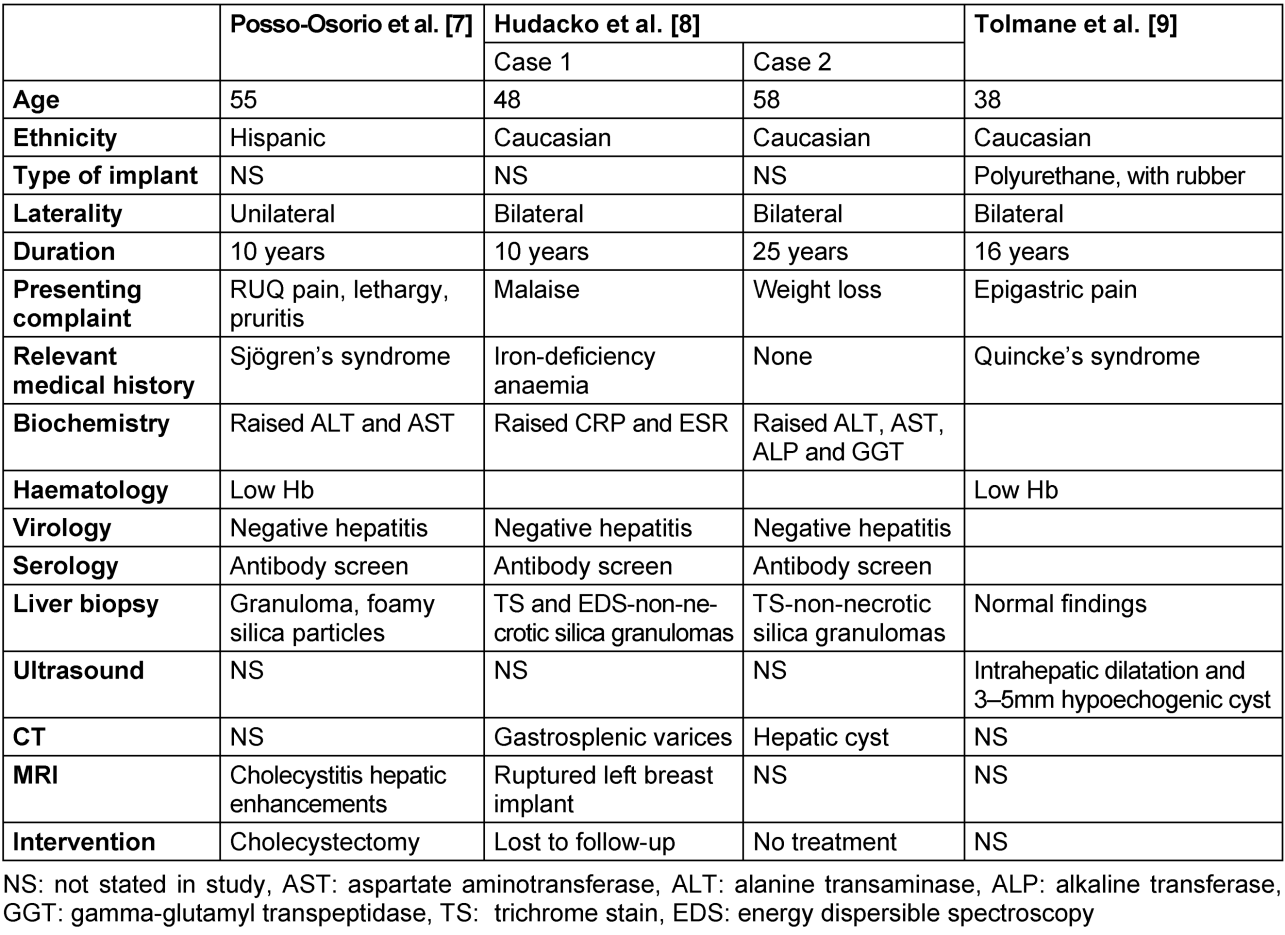
Summary of papers included in final review

## References

[1] Czerny V (1895). Plastic replacement of the breast with a lipoma. Chir Kong Verhandl.

[2] Edgerton MT, McClary AR (1958). Augmentation mammaplasty; psychiatric implications and surgical indications. Plast Reconstr Surg Transplant Bull.

[3] Cronin TD, Gerow FJ (1964). Augmentation mammoplasty: A new ‘natural feel’ prosthesis. Transections of the Third International Congress of Plastic Surgery, Amsterdam. Excerpta Medical.

[4] Necchi S, Molina D, Turri S, Rossetto F, Rietjens M, Pennati G (2011). Failure of silicone gel breast implants: is the mechanical weakening due to shell swelling a significant cause of prostheses rupture?. J Mech Behav Biomed Mater.

[5] Jones JL, Hanby AM, Wells C, Calaminici M, Johnson L, Turton P, Deb R, Provenzano E, Shaaban A, Ellis IO, Pinder SE, National Co-ordinating Committee of Breast Pathology (2019). Breast implant-associated anaplastic large cell lymphoma (BIA-ALCL): an overview of presentation and pathogenesis and guidelines for pathological diagnosis and management. Histopathology.

[6] Frame J (2017). The waterfall effect in breast augmentation. Gland Surg.

[7] Posso-Osorio I, Méndez-Rayo T, Jimenez CA, Escobar D, Sepúlveda M, Navarro EP, Tobón GJ (2018). Hepatic infiltration by silicone in a patient With ASIA syndrome. Hepatology.

[8] Hudacko R, Anand K, Gordon R, John T, Catalano C, Zaldana F, Katz HJ, Fyfe B, Rustgi V (2019). Hepatic Silicone Granulomas Secondary to Ruptured Breast Implants: A Report of Two Cases. Case Reports Hepatol.

[9] Tolmane I, Rozentale B, Keiss J, Putnins V (2011). Liver Damage after Breast Plastic Surgery – Clinical Case Report. Acta Chir Latv.

[10] Moher D, Liberati A, Tetzlaff J, Altman DG, PRISMA Group (2009). Preferred reporting items for systematic reviews and meta-analyses: the PRISMA statement. PLoS Med.

[11] The Joanna Briggs Institute (2014). The Joanna Briggs Institute Reviewer’s Manual 2014 – The Systematic Review of Prevalence and Incidence Data.

[12] Park AJ, Chetty U, Watson AC (1996). Patient satisfaction following insertion of silicone breast implants. Br J Plast Surg.

[13] Lee Y, Song SE, Yoon ES, Bae JW, Jung SP (2017). Extensive silicone lymphadenopathy after breast implant insertion mimicking malignant lymphadenopathy. Ann Surg Treat Res.

[14] Collis N, Litherland J, Enion D, Sharpe DT (2007). Magnetic resonance imaging and explantation investigation of long-term silicone gel implant integrity. Plast Reconstr Surg.

[15] Shoenfeld Y, Agmon-Levin N (2011). ‘ASIA’ – autoimmune/inflammatory syndrome induced by adjuvants. J Autoimmun.

[16] Teuber SS, Howell LP, Yoshida SH, Gershwin ME (1994). Remission of sarcoidosis following removal of silicone gel breast implants. Int Arch Allergy Immunol.

[17] Brandon HJ, Taylor ML, Powell TE, Walker PS (2006). Morphology of breast implant fold flaw failure. J Long Term Eff Med Implants.

[18] Spear SL, Murphy DK, Allergan Silicone Breast Implant U (2014). S. Core Clinical Study Group. Natrelle round silicone breast implants: Core Study results at 10 years. Plast Reconstr Surg.

[19] Dowden RV (1993). Detection of gel implant rupture: a clinical test. Plast Reconstr Surg.

[20] Hölmich LR, Fryzek JP, Kjøller K, Breiting VB, Jørgensen A, Krag C, McLaughlin JK (2005). The diagnosis of silicone breast-implant rupture: clinical findings compared with findings at magnetic resonance imaging. Ann Plast Surg.

[21] Everson LI, Parantainen H, Detlie T, Stillman AE, Olson PN, Landis G, Foshager MC, Cunningham B, Griffiths HJ (1994). Diagnosis of breast implant rupture: imaging findings and relative efficacies of imaging techniques. AJR Am J Roentgenol.

[22] U.S. Food & Drug Administration Silicone-Filled Breast Implants.

[23] DeBruhl ND, Gorczyca DP, Ahn CY, Shaw WW, Bassett LW (1993). Silicone breast implants: US evaluation. Radiology.

[24] Berger D, Desai V, Janardhan S (2019). Con: Liver Biopsy Remains the Gold Standard to Evaluate Fibrosis in Patients With Nonalcoholic Fatty Liver Disease. Clin Liver Dis (Hoboken).

[25] Lo RC, Kim H (2017). Histopathological evaluation of liver fibrosis and cirrhosis regression. Clin Mol Hepatol.

